# Late onset cerebellar ataxia syndrome after non-paraneoplastic Lambert-Eaton myasthenic syndrome: a case study

**DOI:** 10.1186/s12883-024-03983-8

**Published:** 2025-01-02

**Authors:** David P. Randall, Matthew C. Randall

**Affiliations:** 1 Neuromuscular Neurology, Advocate Health, 1850 Dempster Street, Park Ridge, IL 60068, USA; 2 Present Address: Norton College of Medicine, Upstate Medical University, Syracuse, NY USA

**Keywords:** Lambert-Eaton myasthenic syndrome, Cerebellar ataxia, Voltage-gated calcium channel antibodies, Paraneoplastic cerebellar syndrome

## Abstract

This is an unusual case of voltage gated calcium channel (VGCC) antibodies leading to two distinct and chronologically separated neurologic syndromes without the presence of an underlying neoplasm. Lambert Eaton Myasthenic Syndrome (LEMS) presented five years prior to cerebellar ataxia. Both LEMS and cerebellar ataxia were responsive to treatment, but not the same therapy. He was diagnosed with LEMS through history, exam, electromyography/nerve conduction studies (EMG/NCS) with repetitive nerve stimulation (RNS) and antibody testing. He was treated with 3,4 diaminopyridine (3,4 DAP) with an excellent response. Five years later, he developed acute ataxia. The patient required months of intensive and continued immunomodulating therapy.

## Case

A 56 year-old right handed man presented to the Neuromuscular Clinic with a two month history of difficulty playing the drums and singing in his band. Initially he noted progressive fatigue and weakness with repetitive actions. Just 1 week later he noted trouble walking upstairs, getting out of chairs and climbing a ladder. His shoulders became weak. Over a few weeks he developed dysarthria and a dry mouth. He denied pain, numbness, tingling, double vision, ptosis, trouble with chewing, swallowing, or shortness of breath. He had been a 1–2 pack per day (ppd) smoker for 40 years. Neurologic exam showed normal mental status and cranial nerves. He had proximal weakness in the legs more than the arms but excellent distal power. Deep tendon reflexes were hypoactive in the arms and absent in the legs. There was a normal sensation to pin. He had no ataxia or tremor and normal rapidly alternating movements. Gait was limited only by leg weakness.

Tests showed creatine phosphokinase (CPK) and myasthenia gravis (MG) antibodies were all within normal limits. The MG antibodies were acetylcholine (ACH) receptor binding antibody, ACH receptor modulating antibody, and anti-striated muscle antibody. There were elevated PQ-VGCC antibodies. EMG/NCV demonstrated on RNS a greater than 100% incremental response at several motor nerves following brief exercise. This was followed by a decremental response which is characteristic of an abnormality of the presynaptic neuromuscular junction. Imaging of the brain and cervical spine were unrevealing. A chest X-ray identified a small nodule in his lung. At the initial presentation, the patient had a Delta - P score of 4/6 (bulbar weakness with speaking and singing, autonomic symptoms including erectile dysfunction, tobacco use, and age of 56) suggesting a very high risk of malignancy (Table 1). However, no malignancy was seen on close follow up with CT chest and PET scans. The small nodule identified on the initial chest X-ray was characterized as an enlarged lymph node on initial and repeated CT and PET scans. Continued imaging through 10 years after diagnosis showed no evidence of malignancy.

**Table 1:** Delta P score

Bulbar weakness	Absent Present	0 1
Erectile Dysfunction	Absent Present	0 1
Loss of weight	< 5% ≥ 5%	0 1
Tobacco use at onset	Absent Present	0 1
Age at onset	< 50 years ≥ 50 years	0 1
Karnofsky Performance Score	70–100 0–60	0 1

Risk of small cell lung cancer (SCLC) increases with each point: With 0 points the risk of SCLC is 0% and with 1 point the risk is 2.6%. This increases to 83.9% for 3 points, 93.5% for 4 points, 96.6% for 5 points, and up to 100% for 6 points (1)

He responded exceedingly well to 3,4 DAP and pyridostigmine. The initial dose of 3,4 DAP was 10 mg 3 times per day and the maintenance dose was 65 mg a day in divided doses. The dose of pyridostigmine was 480 mg a day in 4 doses. He had normal power with minimal intermittent fatigue. He never required immune modulating therapy for LEMS.

More than 5 years after his initial diagnosis of LEMS, he developed acute ataxia. Over the course of just 2 weeks he noted trouble walking and his balance was poor. He had several falls. Speech became impaired. He did not feel weak but could not walk or maintain his balance. He developed swallowing difficulty and diplopia. He reported no new medicines, and no preceding immunization or illness. Exam demonstrated dysconjugate gaze, pendular nystagmus, and hypoactive reflexes. He struggled with an intention tremor, scanning speech, dysdiadochokinesia, and an inability to stand or walk without full assistance. He had postural instability even in a chair. He had preserved motor power and sensation.

Work up including MRIs of the brain and the cervical, thoracic and lumbar spine all completed with and without IV contrast, CT chest/abdomen/pelvis and PET scan, all were unremarkable. He was treated with IVIG 2 g/kg x 2 cycles, but continued to progress. He was subsequently treated with high dose IV steroids followed by oral steroids and mycophenolate. Over several months he improved from wheelchair to independent walking with a cane. Vision and speech abnormalities resolved. He had residual mild gait difficulties. He was followed for an additional 5 years after the acute cerebellar ataxia and no cancer was detected during the time of monitoring.

## Discussion

Numerous cases have documented paraneoplastic LEMS and cerebellar ataxia, including in 1957 a patient described by Lambert and Eaton [2]. However it is rarely reported for both LEMS and cerebellar ataxia to occur in a single patient without an underlying cancer [3–5]. This is the first report of a symptomatic non-paraneoplastic LEMS that was followed more than 5 years later by an acute cerebellar syndrome.

LEMS is a rare autoimmune-mediated disorder associated with P/Q-type VGCC antibodies [3]. These antibodies lead to a reduction in presynaptic release of acetylcholine which is the cause of the characteristic clinical expression of proximal weakness, fatigue, reduced tendon reflexes, and associated autonomic dysfunction (Table 2) [6, 7]. The diagnosis of LEMS is made of clinical features and confirmation with electrodiagnostic studies and the detection of P/Q-VGCC antibodies [3, 6].

**Table 2:** Symptoms and signs of LEMS and cerebellar ataxia [6, 8]

Symptom	LEMS	Cerebellar ataxia
Proximal leg weakness	Yes	No
Dry Mouth	Yes	No
Diplopia	Late	Early
Gait Abnormality	Often	Yes
Limb ataxia	No	Yes
Truncal Ataxia	No	Yes
VGCC Abs	Yes > 85%	Infrequent
Neurophysiologic Testing	Abnormal presynaptic neuromuscular transmission.	Normal

A comprehensive EMG/NCV, including RNS, demonstrates the abnormality at the presynaptic neuromuscular junction.

In 85–91% of LEMs cases, P/Q-VGCC antibodies are present and only 10–15% of LEMs patients have no detectable P/Q-VGCC antibodies [7, 9]. This could be explained by an undetectably low concentration of these antibodies or the presence of antibodies to another protein that leads to a similar presentation [7].

Rapidly progressive cerebellar syndrome typically presents with a progressive gait disorder and incoordination [7, 8]. Patients tend to develop both truncal and limb involvement and diplopia as the disease progresses. Numerous antibodies associated with cerebellar ataxia that may be related to cancer have been identified. These include anti-Yo (PCA1), anti-Hu, PCA2, anti-Tr (DNER), anti-SOX1, and anti-Ri (ANNA-2) [8, 10]. VGCC antibodies are a rare cause of autoimmune ataxia [7]. Early treatment of VGCC cerebellar ataxia is more likely to prevent permanent neurologic deficit from purkinje cell death [10, 11].

About half of the cases of LEMS are associated with neoplasia, most often small cell lung cancer (SCLC), which also expresses a functional VGCC [3, 7]. Interestingly, patients with SCLC and associated LEMS live longer than similar stage SCLC patients without LEMS. This may be explained by a stronger immune response against the cancer [6]. Several cancers, including SCLC, are associated with cerebellar degradation due to antibodies against VGCCs in purkinje cells [10, 11]. Cerebellar ataxia is only rarely present with non-paraneoplastic LEMS [4]. Treatment of cerebellar ataxia is typically focused on treating the immune response, and evaluating for cancer. The optimal immune treatment strategy has yet to be determined, but based on other immune mediated cerebellar syndromes early treatment appears to be essential in recovery [2].

Practical Implications: There are now easily accessible antibody studies and effective treatments for LEMS which will lead to an increase in testing, diagnosis and treatment. This study highlights the need to be aware of a secondary less common syndrome of cerebellar ataxia. Recognition and earlier treatment of cerebellar ataxia may help reduce permanent neurologic injury.

## Conclusion

This is a very rare case of VGCC antibodies producing two distinct clinical syndromes more than 5 years apart in a patient without malignancy. With the advent of an effective FDA approved medication for LEMS and a reasonably sensitive and specific antibody test, there will be greater recognition of LEMS, and there are likely to be more patients diagnosed with a second VGCC antibody related syndrome. This case highlights multiple presentations of the VGCC antibody but does not reveal either the trigger of the second syndrome or treatments that could prevent the onset of cerebellar ataxia. Recognizing the variable and complicated presentation of VGCC will help to allow for earlier diagnosis and treatment to prevent permanent cerebellar degeneration.

## Data Availability

No datasets were generated or analysed during the current study.

## Abbreviations

3,4 DAP: 3,4 Diaminopyridine
ACH: Acetylcholine
CPK: Creatine Phosphokinase
EMG/NCS: Electromyography/Nerve Conduction Studies
IVIG: Intravenous Immunoglobulins
LEMS: Lambert Eaton Myasthenic Syndrome
MG: Myasthenia Gravis
PPD: Pack Per Day
RNS: Repetitive Nerve Stimulation
SCLC: Small Cell Lung Cancer
VGCC: Voltage Gated Calcium Channel
